# Supplementary material for: Ag2O-Containing Biocidal Interpolyelectrolyte Complexes on Glass Surfaces—Adhesive Properties of the Coatings
Source: Polymers (Basel). 2023 Dec 13;15(24):4690. doi: 10.3390/polym15244690 (PMC10747383; doi:10.3390/polym15244690)
Supplement: Supplementary file 1 [file polymers-15-04690-s001.zip › polymers-2702570-supplementary.pdf]

Supplementary materials
For

Ag_2O-Containing Biocidal Interpolyelectrolyte Complexes on Glass Surfaces—Adhesive Properties of the Coatings

Vladislava A. Pigareva [1,2], Oleg S. Paltsev [1], Valeria I. Marina [1,3], Dmitrii A. Lukianov [1,3], Andrei V. Moiseenko [4], Nikita M. Shchelkunov [5], Andrey A. Fedyanin [5] and Andrey V. Sybachin [1,*]

1 Faculty of Chemistry, Lomonosov Moscow State University, Leninskie Gory, 1-3, 119991 Moscow, Russia; vla_dislava@mail.ru (V.A.P.); ymmo@mail.ru (V.I.M.); dmitrii.a.lukianov@gmail.com (D.A.L.)
2 A.N. Nesmeyanov Institute of Organoelement Compounds of Russian Academy of Sciences, Vavilov Street, 28, 119991 Moscow, Russia
3 Skolkovo Institute of Science and Technology, Center for Molecular and Cellular Biology, Bolshoy Boulevard, 30, 121205 Moscow, Russia
4 Faculty of Biology, Lomonosov Moscow State University, Leninskie Gory, 1-5, 119991 Moscow, Russia; postmoiseenko@gmail.com
5 Faculty of Physics, Lomonosov Moscow State University, Leninskie Gory, 1-2, 119991 Moscow, Russia; shchelkunov@nanolab.phys.msu.ru (N.M.S.); fedyanin@nanolab.phys.msu.ru (A.A.F.)
* Correspondence: sybatchin@mail.ru

Drop test for bacterial growth inhibition.

The 2 μl aliquots of PDADMAC (20 mg/ml), IPEC (20 mg/ml) and IPEC/Ag2O (20 mg/ml) were dropped on agar plate surface covered with *E. coli K12* strain. In addition an empty glass without polymers coatings was also applied on agar surface. Image of overnight culture is present on Figure S1.

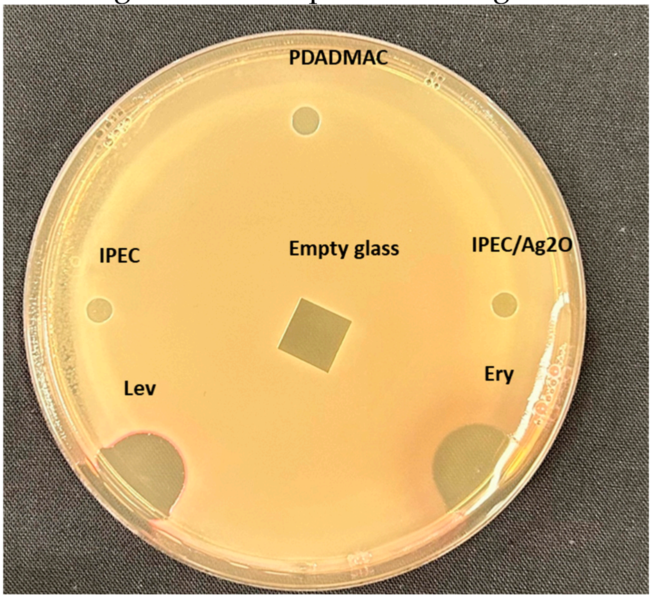

Figure S1. The results of drop test on agar plate with *E. coli K12* strain. Erythromycin (Ery) and levofloxacin (Lev) were used as controls.

It is seen that empty glass without a polymer coating does not provide an inhibition zone

Screening for bacterial growth inhibition.

Glass substrates coated with PDADMAC,IPEC and IPEC/Ag2O were immersed in agar plate surface covered with *E. coli K12* strain. The results of screening

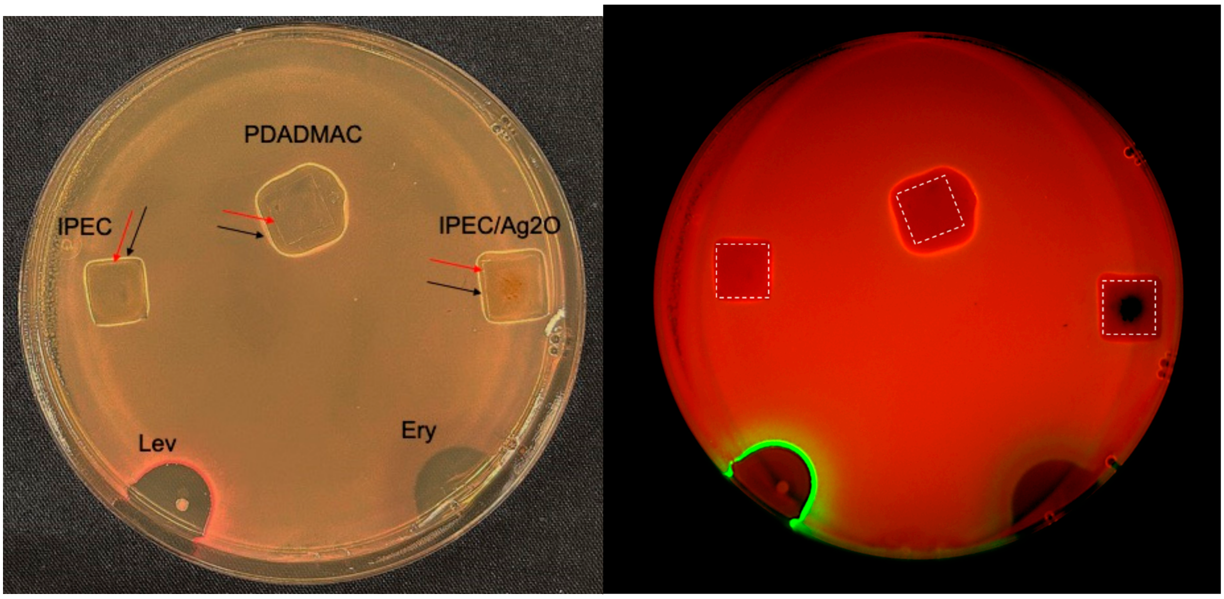

(a) (**b**)

Figure S2. Image of the agar plate after overnight incubation with PDADMAC, IPEC and IPEC/Ag_2O. Black arrows show the boundary of the inhibition zone, red arrows show the boundary of the glasses on which the polymer complexes were applied (**a**); plate scanned in the "cy3-blot" and "cy5-blot" channels (**b**) – the. Erythromycin (Ery) and levofloxacin (Lev) were used as controls.

Estimation of surface roughness of the coatings.

The surface relief of the PDADMAC and IPEC coatings on the glass surface was studied by scanning probe microscope. The typical profiles of the coatings are presented on the Figure S3.

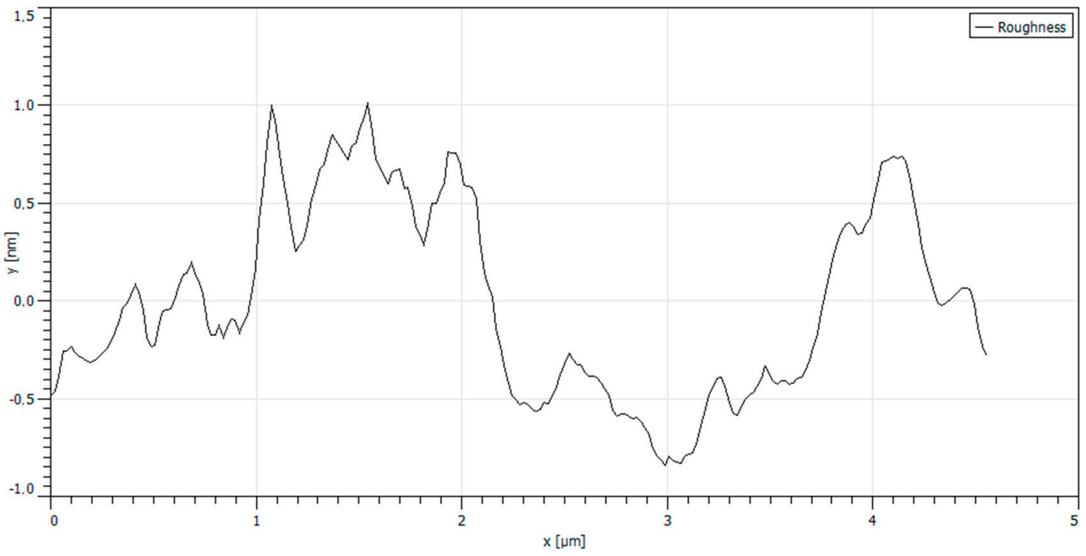

(**a**)

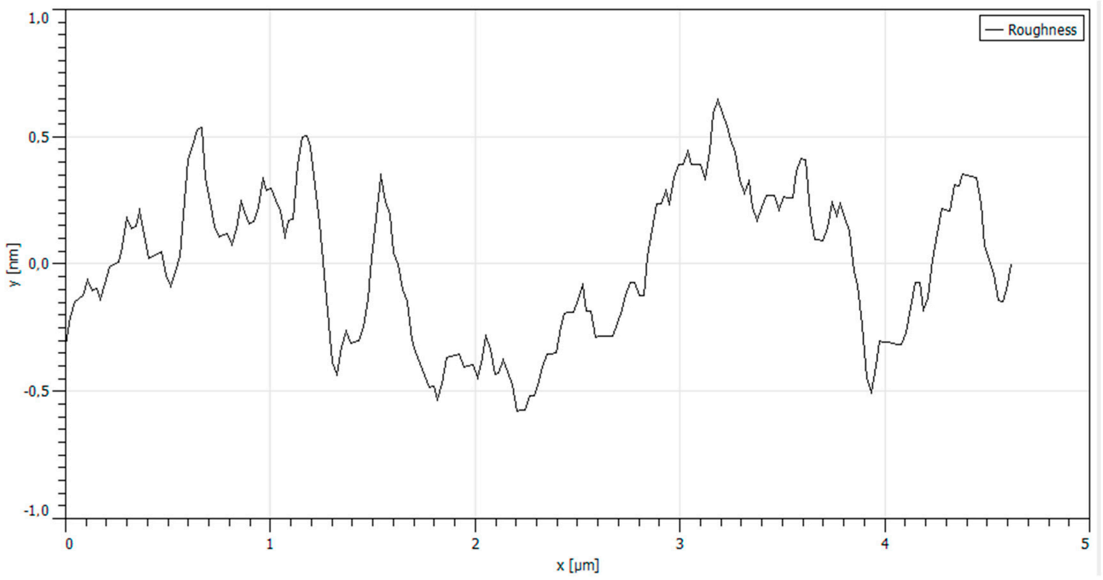

(**b**)

Figure S3. Surface profiles for PDADMAC (**a**) and IPEC (**b**) coatings on glass substrate.
